# Preoperative Surgical Site Hair Removal for Elective Abdominal Surgery: Does It Have Impact on Surgical Site Infection

**DOI:** 10.1055/s-0042-1749425

**Published:** 2022-08-02

**Authors:** Suchin Dhamnaskar, Sumit Mandal, Mandar Koranne, Pratik Patil

**Affiliations:** 1 Seth G.S. Medical College, King Edward memorial hospital, Mumbai, India

**Keywords:** preoperative hair shaving, surgical site infection, elective abdominal surgery

## Abstract

**Introduction** Postoperative surgical site infection (SSI) forms the major burden of nosocomial infections in surgical patients. There is prevalent practice of surgical site hair shaving as a part of preoperative preparation. There is uncertainty regarding the benefit versus harm of shaving for SSIs. Hairs at surgical sites are removed prior to surgery most often by shaving. We performed this study to look for what impact preoperative hair removal by shaving has on postoperative SSI.

**Methods** We performed prospective comparative cohort study in patients undergoing elective abdominal surgeries. We included clean and clean-contaminated surgeries in immunocompetent patients of which half were shaved and other half not shaved prior to surgery. Other confounding factors like skin cleaning, aseptic technique of surgery, antibiotic prophylaxis and treatment, and postoperative wound care were as per care. Patients were assessed for presence and grade of SSI postoperatively on day 7, 14, and 30. Results were analyzed statistically using chi-square and Fischer's exact tests for significance in entire sample as well as in demographic subgroups.

**Results** Overall SSI rate was 11.42%. There was no statistically significant difference in SSI rates between patients who underwent preoperative surgical site hair removal by shaving (232) and who did not have shaving (232) on all the three different assessment timelines in postoperative period, namely, day 7, 14, and 30. Although the absolute number of patients who had SSI was more in those who underwent preoperative surgical site hair removal by shaving, the difference was not statistically significant ( *p* > 0.05). But on subgroup analysis patients with clean-contaminated surgeries ( *p* = 0.037) and patients with surgeries lasting for less than 2 hours (Fischer's exact = 0.034) had significantly higher SSI in the shaved group compared with unshaved on day 14.

**Conclusion** As per our results, preoperative shaving did not significantly increase overall SSI except in subgroup of clean-contaminated surgeries and in surgeries of less than 2 hours' duration. So especially in these patients avoiding preoperative surgical site hair shaving may be used as one of the infection control measures.

The overall incidence of surgical site infection (SSI) following abdominal surgeries was 16.3% in a study conducted by Alkaaki et al in 2019. 1 SSIs not only increase health care cost burden and hospital stay but more importantly they also unduly increase morbidity and mortality associated with the surgical procedures. Hairs have often been perceived to be associated with a lack of cleanliness and its removal linked to infection prophylaxis. 2 Various modalities of hair removal include shaving, clipping, and depilating creams. Shaving results in microscopic cuts and abrasions thus acting as disruption of skin's defense barrier against microorganism colonization. Differences exist about the beneficial vis-a-vis harmful role of shaving in preventing SSI. The Centers for Disease Control and Prevention (CDC) suggested that hair need not to be removed unless it is of surgery, antibiotic prophylaxis and treatment and postoperative wound care were as per will interfere with the operation, and if hair is to be removed it is done immediately before the operation but not in the operation theater itself, with electrical clippers rather than shaving. 3 The Norwegian Knowledge Centre for Health Services could not find evidence against hair removal. 4 The British Hospital Infection Society Working Party guidelines advice shaving only the site of incision. 5 Multiple studies could not find sufficient and conclusive evidence for or against preoperative shaving in preventing SSI. 6 7 8 9 Despite other studies reporting not to remove hair preoperatively 10 11 12 13 unless it interferes with the surgery, many surgeons continue to practice routine preoperative shaving since long as a tradition. We evaluated impact of preoperative hair removal at our teaching hospital setting for clean and clean-contaminated surgeries.

## Aim

To evaluate the effect of preoperative surgical site hair shaving on SSI.

## Objectives

1. To find out the incidence of SSI in patients undergoing preoperative hair removal and those not, and compare them with standard statistical measures.
2. To compare the grades of infection in infected patients by Southampton wound scoring system.
3. To study the effect of demographic variables on the incidence of SSI.

## Methods

This prospective comparative cohort study was conducted in a tertiary care teaching hospital's general surgical department over a period of 12 months.

### Inclusion Criteria

1. Patients above 18 years of age.
2. Patients undergoing elective abdominal surgery for a valid indication.
3. Patients undergoing surgeries in which wounds were primarily closed and fell into clean and clean-contaminated types of surgery as per following the CDC criteria. 14

▪ Clean surgery is the one in which gastrointestinal, biliary, or genitourinary tracts are not entered, there is no acute inflammation, and there is no breach of aseptic technique.
▪ Clean-contaminated surgery is the one where there is controlled opening of gastrointestinal, biliary, or genitourinary tract with no or minimal spillage and when bile or urine are not infected or when there is minor breach of aseptic technique.

### Exclusion Criteria

1. Pregnant or lactating women.
2. Patients with chronic medical illness, viz. uncontrolled diabetes mellitus (hemoglobin A1c greater than 8).
3. Skin diseases involving the site of proposed incision.
4. Chronic dermatological condition altering healing rate.
5. Wounds left open for healing with secondary intention.
6. Immunocompromised condition impairing wound healing.
7. Collagen vascular disorders.
8. Second laparotomy through the same incision within the follow-up period.
9. Patients on chronic steroid therapy.

### Sample Size Calculation

Sample size calculation was done using the following



Here, percent of unexposed with outcome was 2.4 and percent of exposed with outcome was 8.2. 15

### Study Procedure

Institutional ethics committee approval was obtained before study commencement. All eligible patients were enrolled after informed consent. Group A included patients who had preoperative hair shaving and group B included those whose hairs were not shaved before surgery. (In our department, some consultants prefer preoperative shaving whereas others do not.) All surgeries were performed by qualified consultants with at least 3 years of experience. Shaving was done, immediately prior to the surgery, by the barber appointed by employer. Preoperative optimization, preoperative surgical site preparation, antibiotic prophylaxis, and aseptic precautions were same in both the groups and as per routine standard of care. Postoperative antibiotic, analgesic treatment, as well as wound care were same and as per routine standard of care. Relevant demographic data was noted and entered in predesigned case record forms. SSI in postoperative wound was assessed by the principal investigator alone to avoid interobserver variability, on postoperative days 7, 14, and 30 as per Southampton wound scoring system and data entered in case record forms. Results were compared for statistical significance using chi-square test and Fischer's exact test. Pain during change of dressing on postoperative day 7 was assessed in both the groups as reported by patients on visual analogue scale and the results compared statistically using Mann–Whitney *U* test.

### Southampton Wound Scoring System 16

**Table:** 

Grade	Appearance
0	Normal healing
1	Normal healing with mild bruising or erythema a. Some bruising b. Considerable bruising c. Mild erythema
2	Erythema plus other signs of inflammation a. At one point b. Around sutures c. Along wound d. Around wound
3	Clear or hemoserous discharge a. At one point only (up to 2 cm) b. Along wound (more than 2 cm) c. Large volume d. Prolonged (more than 3 days)
4	Pus a. At one point only (less than 2 cm) b. Along the wound (more than 2 cm)
5	Deep or severe wound infection with or without tissue breakdown; hematoma requiring aspiration

## Results

Fifty-three out of the total sample size of 464 patients (11.42%) had SSI overall. The average age of the study population was 42.47 years. Number of males (307; 66.16%) present were almost twice that of females (157; 33.84%). Clean surgeries were 198 (42.67%) and the rest were clean-contaminated surgeries 266 (57.33%). Average body mass index (BMI) of the study population was 22.88 kg/m ^2^ . Two hundred and five patients underwent laparoscopic surgery (44.18%) and the rest were open surgeries (55.82%). The surgical wound closure was done with skin staplers in 2.80% patients (13/464), and with suture material in the rest, monofilament (262/464) being 56.47% and poly filament (189/464) being 40.73%%. Local anesthesia was used during wound closure in 76.08% patients (353/464).

Table 1 shows distribution of both the groups (shaved and unshaved) according to various demographic criteria and the number of patients having SSI in each of these subgroups.

**Table 1:** Demographic subgroups of the study population

Characteristics	Total (464)	Shaved (232)	Not shaved	SSI on day 7 in		SSI on day 14 in		SSI on day 30 in	
			(232)	Shaved	Not shaved	Shaved	Not shaved	Shaved	Not shaved
Gender									
Male	307	225	82	21	9 11	23 2	11 9	12 0	9 4
Female	157	7	150	1					
COC classification									
Clean	198	144	54	9	0 20	8 17	2 18	4 8	1 12
Clean-contaminated	266	88	178	13					
BMI									
< 25	367	186	181	20	15 5	24 1	16 4	12 0	12 1
> 25	97	46	51	2					
Surgery									
Open	259	159	100	21	18 2	25 0	17 3	12 0	10 3
Lap	205	73	132	1					
Duration of surgery									
< 2 h	247	122	125	4	1 14 4 1	7 16 1 1	1 13 4 2	3 8 0 1	0 98 2 2
2–4 h	189	98	91	16					
4–6 h	22	9	13	1					
> 6 h	6	3	3	1					
Wound closure									
Stapler	13	9	4	4	0 18 2	4 17 0	0 21 3	1 11 0	0 12 1
Monofilament suture	262	151	111	17					
Poly-filament suture	189	72	117	1					
Local anesthesia					17 3	19 6	15 5	9 3	11 2
Given	333	171	182	16					
Not given	111	61	50	3					

Abbreviations: BMI, body mass index; CDC, Centers for Disease Control and Prevention; SSI, surgical site infection.

**Fig. 1:**
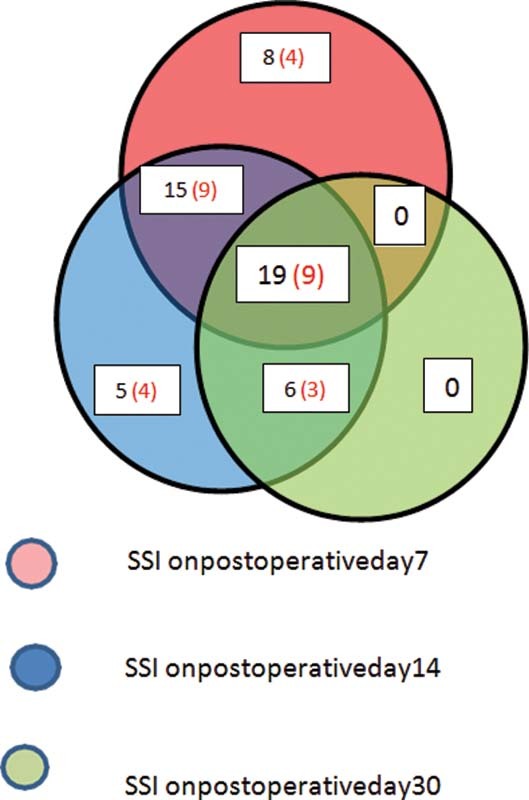
Pictorial representation of distribution patients who had surgical site infection (SSI), with numbers bracketed in red denoting SSI in shaved patients.

Fig. 1 shows temporal distribution of SSI in both the groups (shaved and unshaved) with respect to three timelines of outcome assessment (day 7, 14, and 30). On 7th day assessment, total of 42 (8 + 15 + 19) patients had SSI of which 22 (4 + 9 + 9) were in the shaved group and other 20 in the unshaved group. Of the total of 42 SSI on day 7, a total of 8 (4 shaved group and the rest in the other group) resolved before next assessment on day 14. Of these 42 SSI, 34 (15 + 19) continued to have SSI on day 14. Of these 34 SSI, 15 resolved before day 30 whereas19 SSI were continued even till day 30. Total of 45 (15 + 5 + 19 + 6) patients had SSI on day 14, of which 34 (15 + 19) were those who had SSI since day 7. And of these 34, 15 resolved before day 30. Total of 11 (5 + 6) new SSI were found on day 14 of which 5 resolved before day 30 and 6 continued on day 30. On day 30, total of 25 (19 + 6) SSI were seen. Of which 19 had continued right from day 7 through day 14 and 6 were those who were detected on day 14 and continued to have SSI on day 30. No new SSI were found on day 30.

There was no statistically significant difference in SSI rates between patients who underwent preoperative surgical site shaving and those who did not have shaving on all the three different assessment timelines in postoperative period, namely, day 7, 14, and day 30. Although the absolute number of patients who had SSI was more in those who underwent preoperative shaving, the difference was not statistically significant ( *p* > 0.05) ( Table 2 ).

**Table 2:** SSI on postoperative day 7, 14, and 30

	Postoperative day 7	Postoperative day 14	Postoperative day 30
Preoperatively hair shaved	22	25	12
Preoperatively hair *not* shaved	20	20	13
Statistical significance	(chi-square = 0.015, *p* = 0.75)	(chi-square = 0.615, *p* = 0.43)	(chi-square = 0.042, *p* = 0.83)
SSI (overall)	42 (9.05%)	45 (9.25%)	25 (5.39%)

Abbreviation: SSI, surgical site infection.

For the purpose of statistical comparison, Southampton wound score of postoperative SSI was grouped to make two grades, namely, Minor SSI (scores 1 and 2) and Major SSI (scores of 3, 4, or 5). On comparing these grades of SSI between shaved and unshaved patients there was no significant difference in the rates of SSI ( Table 3 ) on day 7, 14, and 30. *p* -Value was greater than 0.05 on all assessment times.

**Table 3:** Grades of infection in shaved and unshaved patients with their statistical comparison

Grouped Southampton wound scores	Postoperative day 7		Postoperative day 14		Postoperative day 30	
	Shaved	Not shaved	Shaved	Not shaved	Shaved	Not shaved
Minor Infection (1 and 2)	11	9	14	8	4	1
Major Infection (3, 4, and 5)	11	11	11	12	8	12
Statistical significance	Not significant (chi-square = 0.105, *p* = 0.75)		Not significant (chi-square = 1.138, *p* = 0.28)		Not significant (Fisher's exact = 0.1602)	

**Chart 1:**
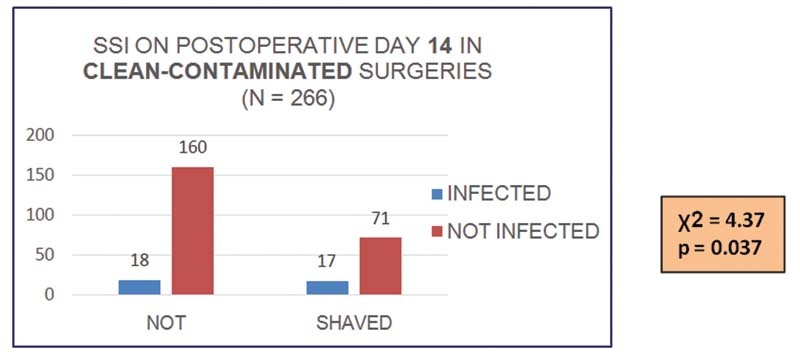
Surgical site infection (SSI) on postoperative day 14 in clean-contaminated surgeries.

On subgroup analysis, clean-contaminated surgeries had significantly more SSI in shaved patients on postoperative day 14 ( *p* = 0.037) ( Chart 1 ). However, this difference was not observed in clean surgeries or on postoperative days 7 and 30 in either type of surgery. Among 266 clean-contaminated surgeries, 35 (13.16%) were infected on postoperative day 14.

**Chart 2:**
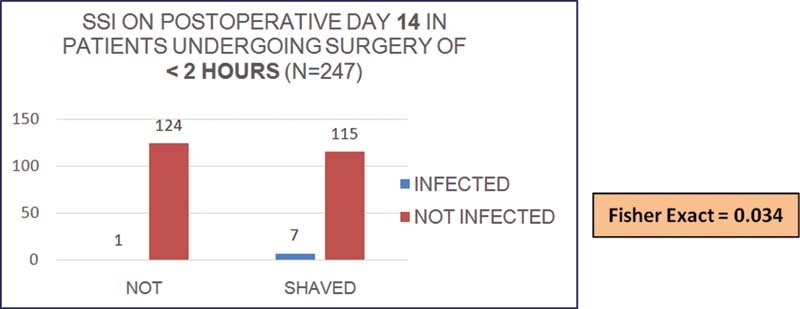
Surgical site infection (SSI) on postoperative day 14 in short surgeries.

Short surgeries of less than 2 hours' duration had significantly more SSI in the shaved patients compared with unshaved patients on postoperative day 14 ( Chart 2 ). Such a difference was not observed in longer surgeries of more than 2 hours' duration nor on any other postoperative days.

There was no significant difference in the pain caused by the change of dressing in shaved and unshaved patients. The amount of pain during change of dressing was measured with visual analogue scale and the pain was graded as *minimal* , *mild* , *moderate* , *significant* , and *severe* . When the number of patients in each of these grades was compared statistically there was no significant difference between patients who had shaving and who were not shaved. (The *U* -value is 11.5. The critical value of *U* at *p* < 0.05 is 4. Therefore, the result is *not* significant at *p* <05.) chi-square = 2.43, *p* = 0.66.

No statistically significant difference in SSI was found between shaved and unshaved patients when other demographic subgroups were compared (gender, BMI, clean sur- geries, laparoscopic vs. open surgeries, surgeries of more than 2 hours' duration, suture material used for wound closure, and administration of local anesthesia).

## Discussion

Traditionally, hair removal at the surgical site has been considered as a mandatory requirement prior to not only elective but even an emergency surgery. Excessive hairs have been considered unhygienic and associated with uncleanliness. Thus, hair at the surgical site has been linked to infections. And these preconceptions form the basis of long-standing practice of preoperative hair removal. Method and timing of preoperative hair removal has been studied. Various methods of hair removal include shaving, depilatory creams, and electric clipping. Of these shaving is the most commonly practiced in most of the resource-constraint setting like ours. It has been recommended that if one opts for preoperative hair removal it should be done just prior to surgery but not in the operation theater. But this timing is not strictly adhered to at many centers due to logistic reasons. Overall SSI rate in our study was 11.42%. For clean surgeries it was (12/198) 6.06% and that for clean contaminated was (41/ 266) 15.41%. It is comparable to some of the notable studies as follows.

Weiss et al (U.S.) 17 found SSI rate of 2.6% for clean wounds, 3.6% for clean-contaminated wounds, and 10.5% for contaminated and dirty wounds. Hernandez et al (Peru) 18 had overall SSI rate of 26.7%, subgroup results being 13.9% for clean and 15.9% for clean-contaminated. Study by Brown et al (Russia) 19 had overall 9.5% SSI. Arabshahi and Koohpayezade (Tehran) 20 = overall 8.4%; Kaya et al (Turkey) 21 = overall 8.8%; Petrosillo et al (Italy) 22 = overall 5.4% (clean = 3.4%; clean-contaminated = 5.2%; contaminated = 9.8%; dirty = 28%); Rocha-Almazán et al (Spain) 23 = overall 5%; Fusco S de et al (Brazil) 24 = 11.9%; Fiorio et al (Italy) 25 = 11.9%; and Ahmed et al (Pakistan) 26 = overall 11% (clean = 7.2%; clean-contaminated = 19.4%). Studies from India too also show a similar SSI rate: Murty and colleagues 27 = 13%; Lilani et al 28 = overall 8.95% (clean = 3.01%; clean-contaminated = 22.4%); and Ajaz Mustafa et al (Kashmir) 29 = overall 13%.

When both the study groups were divided into subgroups according to duration of surgeries like less than 2 hour, 2 to 4 hour, 4 to 6 hour, and more than 6 hour groups and compared statistically, no significant difference was found in incidence of SSI, *except in cases of short surgeries of less than 2-hour duration, where SSI was significantly more on postoperative day 14 in those patients who underwent preoperative shaving compared with those who did not undergo shaving* . The risk of SSI increases with duration of surgery. 30 Factors which are responsible for this include prolonged exposure to the environment, increased blood loss, prolonged hypothermia, declining levels of antibiotics, etc. In fact, the duration is such an important factor that it is incorporated in the U.S. National Nosocomial Infections Surveillance risk stratification system. In our study, there was an increasing trend of infection as the duration of surgeries increased, but for a given duration, it did not differ significantly whether hair were shaved or not.

According to our results, there was no statistically significant difference in overall SSI rates between patients who underwent preoperative shaving versus those who did not. Quite a few previous studies in the past have shown that shaving caused increase in SSI. 31 32 33 34 35 The procedure of shaving the operation site with a sharp blade may result in abrasions at skin surfaces with bacteria getting lodged in these abrasions which act as foci of infection. 36 The serum which oozes out and gets collected at the sites of these abrasions provide favorable culture media for growth of these organisms and promote SSI. 37 38 39 Contrary to that, many reviews 5 7 and studies 8 have found that evidence for or against hair removal to reduce SSI rates is inconclusive and insufficient. Review of previous studies done by Tanner et al 40 found no statistically significant effect of hair removal on SSI rates. This was similar to our results which also showed no significant difference of SSI.

Nonsignificant effect of shaving was maintained on subgroup analysis as per sex. Such a subgroup analysis was not conducted in any study earlier. Previous studies have compared SSI rates in males versus females and found variable results. Some of them reported more incidence in females, 41 some showed more SSI rates in males, 42 and some reported no effect of sex on SSI. 43 44 There was no significant difference in SSI among patients who were shaved preoperatively and who were not shaved, irrespective of their BMI. Although it is known that risk of SSI increases with increase of BMI from several studies, 20 25 45 this difference was not observed in our study.

Choice of material to close the surgical wound did not lead to any statistically significant difference in SSI. When wounds closed with skin staplers, monofilament sutures, and poly-filament sutures were compared separately statistically, there was no significant difference in SSI on postoperative days 7, 14, or 30 between groups of patients shaved and not shaved. Although staplers are superior in terms of time taken 46 47 to close the incisions, the rate of SSI had been found to be more in few studies. 46 48 But we did not find significant difference in SSI between shaved and unshaved patients on subgroup analysis irrespective of whether the skin was closed with stapler, monofilament, or poly-filament suture materials.

**Chart 3:**
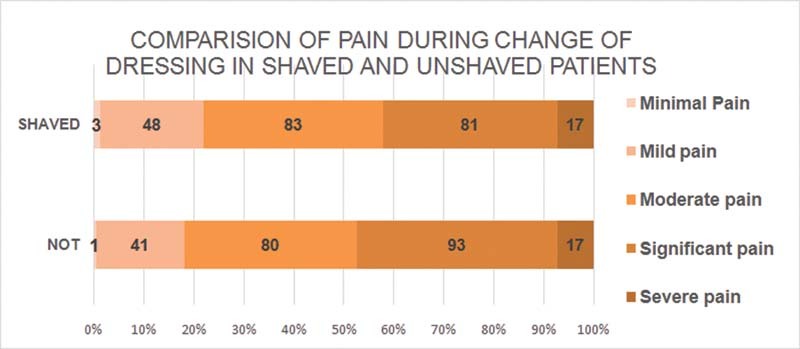
Comparison of pain during change of dressing in shaved and unshaved patients.

Local anesthetic infiltration is associated with a lower incidence of SSI. 49 There was no difference of SSI in patients who underwent preoperative surgical site shaving and who did not irrespective of administration of local anesthesia. There was no significant difference in SSI in shaved and unshaved patients, irrespective of whether laparoscopic or open procedure was performed. Previous studies 50 51 52 found that laparoscopic surgeries had fewer SSI complication rates than open, but this difference was not demonstrated in our study. Nonshaving especially in hairy patients not only may make surgery look a little clumsy due to interference by hair at surgical incision site but also poses a peculiar issue related to wound care and dressing change postoperatively. While changing the dressing when adhesive tapes applied to the dressing to hold it in place are removed, it causes uprooting of intact hair follicles and results in pain and minor injuries. This may even lead to folliculitis. This also results in increased pain during change of dressing. But contrary to the expectation in our study there was no statistically significant difference between the pain caused by the change of dressing in shaved patients and unshaved patients ( chart 3 ). Single observer recording outcomes in all patients to avoid interobserver variability and prospective type of study design are the strengths and observational noninterventional design was the limitation.

## Conclusion

Thus, as per results of our study, though shaving resulted in more SSI in some specific subgroups like clean-contaminated surgeries and in surgeries lasting for less than 2 hours' duration as on postoperative day 14, overall difference in SSI among both shaved and unshaved patients were not statistically significant. So, we conclude that preoperative shaving does not alter SSI. But avoiding shaving of surgical site prior to abdominal surgery may be utilized as one of the measures of reducing occurrence of postoperative SSI especially in clean-contaminated surgeries and short surgeries of less than 2 hours' duration.
